# Differential effects of obstructive sleep apnea on the corneal subbasal nerve plexus and retinal nerve fiber layer

**DOI:** 10.1371/journal.pone.0266483

**Published:** 2022-06-30

**Authors:** Katherine A. Bussan, Whitney L. Stuard, Natalia Mussi, Won Lee, Jess T. Whitson, Yacine Issioui, Ashley A. Rowe, Katherine J. Wert, Danielle M. Robertson

**Affiliations:** 1 Department of Ophthalmology, The University of Texas Southwestern Medical Center, Dallas, TX, United States of America; 2 Department of Internal Medicine, Clinical Center for Sleep and Breathing Disorders, The University of Texas Southwestern Medical Center, Dallas, TX, United States of America; Weill Cornell Medicine-Qatar, QATAR

## Abstract

**Purpose:**

Obstructive sleep apnea (OSA) is an established independent risk factor for peripheral neuropathy. Macro and microvascular changes have been documented in OSA, including high levels of potent vasoconstrictors. In diabetes, vasoconstriction has been identified as an underlying risk factor for corneal neuropathy. This study sought to establish a potential relationship between OSA and corneal nerve morphology and sensitivity, and to determine whether changes in corneal nerves may be reflective of OSA severity.

**Design:**

Single center cross-sectional study.

**Methods:**

Sixty-seven patients were stratified into two groups: those with OSA and healthy controls. Groups were matched for age, sex, race, smoking, and dry eye status. Outcome measures included serologies, a dilated fundus exam, dry eye testing, anthropometric parameters, corneal sensitivity, subbasal nerve plexus morphology, retinal nerve fiber layer (RNFL) thickness, and the use of questionnaires to assess symptoms of dry eye disease, risk of OSA, and continuous positive airway pressure (CPAP) compliance.

**Results:**

No significant differences were observed in corneal nerve morphology, sensitivity, or the number of dendritic cells. In the OSA test group, RNFL thinning was noted in the superior and inferior regions of the optic disc and peripapillary region. A greater proportion of participants in the OSA group required a subsequent evaluation for glaucoma than in the control. In those with OSA, an increase in the apnea hypopnea index was associated with an increase in optic nerve cupping.

**Conclusions:**

OSA does not exert a robust effect on corneal nerves. OSA is however, associated with thinning of the RNFL. Participants with glaucomatous optic nerve changes and risk factors for OSA should be examined as uncontrolled OSA may exacerbate glaucoma progression.

## Introduction

Obstructive sleep apnea (OSA) is a sleep disorder characterized by recurrent pharyngeal collapse resulting in episodic apneas and hypopneas. The overall estimate of OSA in the general population ranges from 9% up to 38% and is more common in men than pre-menopausal women [1–4]. For both men and women, obesity significantly increases the prevalence of this disease [3]. Recurrent hypoxia with reactive oxygen species production underlies vascular endothelial damage and vascular dysregulation [5]. Intermittent nocturnal hypoxia has also been shown to result in a decrease in sural sensory nerve action potentials [6]. Consistent with this, OSA has been shown to alter visual evoked potentials in the eye [6–10]. Clinically, ocular neuropathic manifestations of OSA include non-arteritic anterior ischemic optic neuropathy, papilledema, and glaucoma [11–14]. Apart from optic nerve pathology, OSA is associated with keratoconus and floppy eyelid syndrome [15–18]. Despite corrective surgery for floppy eyelid syndrome, clinical observations suggest that the ocular surface remains abnormal. Not surprisingly, dry eye disease has also been linked with OSA, but corneal damage is largely attributed to air leakage from continuous positive airway pressure (CPAP) use [19].

*In vivo* confocal microscopy (IVCM) is a non-invasive and fully quantitative modality used to study the corneal subbasal nerve plexus (SBNP). The SBNP is located in the cornea between the basal epithelium and Bowman’s layer. Previous studies in our laboratory and others have used IVCM to quantify SBNP changes in diabetes and are reviewed elsewhere [20–25]. These changes include a reduction in nerve fiber density, nerve fiber length, nerve fiber branch density, and increases in tortuosity and dendritic cell infiltration. Early loss of the SBNP however, has been repeatedly demonstrated to occur prior to peripheral nerve damage in diabetes [26–29]. Given the preponderance of evidence, loss of the SBNP is now considered one of the most sensitive early measures of diabetic peripheral neuropathy [25].

The mechanisms that underlie the development of neuropathy are complex and multifactorial. In corneal neuropathy, changes in acetylcholine-mediated relaxation of the posterior ciliary artery have been associated with loss of corneal nerves [30]. Moreover, systemic therapy that promotes artery and arteriole relaxation in diabetes inhibits corneal nerve loss. In OSA, an increase in potent vasoconstrictors such as endothelin, contribute to macro and microvascular damage. Indeed, published data has confirmed thinning of the retinal nerve fiber layer (RNFL) in participants with OSA [31–47]. Similar to peripheral nerves, it is thought that hypoxic damage during sleep and vascular dysregulation drive RNFL thinning in OSA [32, 48]. Despite the large number of studies evaluating the effects of OSA on the posterior eye, the impact of OSA on the cornea and SBNP is unknown. Given the established role of OSA in peripheral neuropathy, the present study sought to examine whether OSA leads to a decrease in corneal sensitivity and abnormal morphological changes in the corneal SBNP. Thickness of the RNFL was further assessed to determine the effects of OSA on the posterior segment of the eye.

## Methods

### Study design

This was a single visit, cross-sectional, masked study to investigate corneal and retinal nerve fiber morphology, cellular changes in the cornea, corneal sensitivity and ocular surface disease in participants with OSA. Study procedures were approved by the institutional review board at the University of Texas Southwestern Medical Center (UTSWMC), Dallas, Texas. This study was performed according to the Declaration of Helsinki for human studies and in accordance to HIPAA regulations. Signed written informed consent was obtained from each patient prior to enrollment in the study. Participants were recruited over a three-and-a-half-year period from 2015 to 2019 through the Department of Ophthalmology and the Clinical Center for Sleep and Breathing Disorders at UTSWMC. All participants were adults, age 18 and older. Participants were stratified into two groups: those with OSA and healthy, non-OSA controls. All participants were matched for age, sex, race, and smoking status. Inclusion criteria for the OSA group included a physician diagnosis of OSA and an overnight polysomnogram within the last five years. To eliminate the confounding effect of CPAP use, only OSA participants that were non-compliant or denied CPAP use were enrolled. CPAP non-compliance was defined as less than four hours per night and was determined using a questionnaire (S1 Fig) [49]. Control participants were defined as healthy non-pregnant adults aged 18 years and older who were not current contact lens wearers and had no history of contact lens wear within the last year. Participants were also excluded if they had a STOP BANG score ≥ 5, since STOP BANG scores 5 and above have been show to identify those with a high risk for moderate to severe OSA [50]. A urine pregnancy test was used to exclude pregnancy in all females of child bearing age without a diagnosis of menopause, tubal ligation or hysterectomy. Additional exclusion criteria for both groups included a history of ocular surgery or trauma within the prior 12 months, active or previous history of herpes virus keratitis, current use of topical glaucoma medications or other topical ophthalmic medications, contact lens wear within the last year, pregnancy or lactation, type 1 or type 2 diabetes mellitus, respiratory disorders such as asthma or chronic obstructive pulmonary disease, end-stage renal disease, alcoholism, infectious disease, currently undergoing treatment for cancer, or any systemic disease that may adversely affect study results.

Participant medical and ocular history was reviewed at the time of visit. This included use of topical and systemic medications. Polysomnograms were obtained from the patient’s electronic medical record or consents were signed to obtain them from the appropriate physicians. The apnea-hypopnea index (AHI) and the lowest oxygen saturation values were recorded. The AHI classification scheme for severity of OSA is based upon the number of apneas and hypopneas that occur per hour of sleep. Less than 5 events per hour is considered none or minimal, 5 to < 15 events per hour signifies mild OSA, 15 ≥ to < 30 is moderate OSA, and ≥ 30 represents severe disease [51]. Oxygen saturation (SpO2) was considered normal if they fell between 96%– 97%, mild if between 80%– 96%, moderate if between 80% - 89%. Anything below 80% was considered severe [52].

For those participants who had abnormal optic nerve findings, a referral was made to the glaucoma clinic for evaluation. Clinic notes from this visit were evaluated to determine whether a diagnosis of glaucoma was confirmed. All other outcome measures were made by a single clinical investigator (DMR). The clinical investigator was masked to the health status of the patient. Outcome measures included the following: completion of the Ocular Surface Disease Index (OSDI) questionnaire, a validated questionnaire to subjectively assess dry eye, as well as a STOP BANG questionnaire to assess risk for OSA. Anthropometric measurements, serology testing and blood pressure measurements were performed on the day of the visit. A complete ophthalmic examination was also performed. This included a dry eye evaluation (described below) and a dilated fundus exam. Participants were dilated by instillation of 1% tropicamide ophthalmic solution (Alcon Laboratories, Ft. Worth, Texas) and 2.5% phenylephrine (Akorn, Lake Forest, IL). Corneal nerves were evaluated using *in vivo* confocal microscopy (IVCM), retinal imaging was performed using optical coherence tomography (OCT), and corneal sensitivity testing using a Cochet-Bonnet aesthesiometer. Specific details are described below.

### Assessment of the cornea and tear film

All ophthalmic examinations were performed as previously described [20]. A biomicroscopic examination of the lids, lashes, conjunctiva, and cornea was performed to rule out any preexisting corneal pathology that could interfere with study findings. Measures of tear film break-up time (TFBUT) and basal tear production were obtained. For TFBUT, 2 μL of 2.0% non-preserved fluorescein (Greenpark Pharmacy, Houston, TX, USA) were instilled onto the superior bulbar conjunctiva. Fluorescein was visualized using a cobalt blue light and a Wratten #12 filter. Following this, participants were asked to blink normally three times. The time between the third blink and the formation of the first dark spot was measured. Three measurements per eye were recorded with 30-second rest periods in between. A 30 second cutoff was used for participants with a stable tear film and no signs of breakup. Immediately following TFBUT measurements, the cornea was evaluated using the National Eye Institute scale for corneal staining [49]. This consisted of grading five different regions in the cornea using a scale of 0 to 3. Finally, a Schirmer’s tear test without anesthesia was performed to look for gross changes in tear production. The Schirmer’s strip was positioned in the lower fornix adjacent to the lateral canthus. Following placement subjects were instructed to close their eyes. After 5 minutes, the length of tear migration on the strip was recorded. All tear film testing was performed on both eyes. The right and left eyes were averaged to achieve a final measurement.

### Corneal sensitivity

The right eye was evaluated for corneal sensitivity using a Cochet-Bonnet esthesiometer (Luneau, Paris, France). The esthesiometer contained a 0.08-mm diameter nylon filament. The fiber was used to applanate the inferior portion of the cornea roughly 2 mm superior to the inferior limbus. Measurements were begun with the filament fully extended to 6.0 mm. This length was systematically decreased in increments of 0.5 mm until the patient could no longer feel >2 of the 4 applanations. False presentations (the filament not actually touching the cornea) were used as a control.

### *In vivo* confocal microscopy

IVCM was performed on each patient using a custom, in-house modified HRT-RCM confocal microscope (Heidelberg Engineering, Heidelberg, Germany) to obtain images of the corneal SBNP and the basal epithelium in the central cornea [53, 54]. The right eye of each patient was used for scanning. Prior to scanning, one drop of 0.5% proparacaine hydrochloride ophthalmic solution (Alcon Laboratories, Ft. Worth, TX, USA) was instilled into both eyes. To optically pair the confocal tip to the cornea, GenTeal Gel (Alcon Laboratories) was placed on the confocal cap. Sequential scans were systematically acquired across the midperipheral and central cornea, beginning in the superior temporal cornea and finishing in the inferior nasal region. Corneal scans were performed in real time and the high-resolution images were then saved as videos to an external hard drive. The objective lens was controlled remotely using a motorized system to adjust focusing throughout the scan. Eight non-overlapping images of the SBNP were extracted from sequential frames. Images were processed and analyzed using MetaMorph (Molecular Devices, Sunnyvale, CA, USA) and FIJI (provided in the public domain by the National Institutes of Health [NIH], Bethesda, MD, USA). Confocal images were analyzed by a single trained investigator (WLS) for determination of nerve fiber length, nerve fiber branch density, and nerve fiber density. Nerve fiber length was defined as the total length in μm of all the nerves in the 400 μm × 400 μm image. This was measured by manually tracing the nerves within each image using MetaMorph software (Molecular Devices). Nerve branch density was defined as the total number of branch points per μm^2^ per image and nerve fiber density was defined as the number of main nerve fibers (not branches) per image. FIJI was used for determination of the number of dendritic cells in each image.

### Assessment of retinal nerve fiber layer

Following the dilated fundus exam, OCT was performed using the Spectralis Tracking Laser Tomography system (Spectralis OCT, Heidelberg Engineering, Heidelberg Germany). Scans were obtained of the optic disc insertion/peripapillary region and the macula. A computerized tracking system accounted for eye movements during the examination.

### Anthropometric measurements

A standard seamstress tape measure was used to obtain measurements of the neck, waist, and hip circumferences of each patient. Neck circumference was obtained by placing the tape around the neck, approximately 1 inch above the intersection of the neck and shoulders. For determination of waist circumference, the tape was placed midway between the lower palpable rib and the iliac crest. Hip circumference was measured around the widest point of the buttocks. Measurements of height and weight were also taken at the study visit. Body mass index (BMI) was calculated based upon the patient’s height and weight measurements. The National Institutes of Health (NIH) body mass scale was used to classify participants as follows: underweight (≤ 18.5), normal weight (18.6–24.9), overweight (25.0–29.9), and obese (≥ 30.0) [55].

### Measurements of systemic health

Systolic and diastolic blood pressure measurements were obtained using an automated wrist sphygmomanometer after the patient had been seated for five minutes. Phlebotomy was performed on fasted participants to determine the levels of glycosylated hemoglobin (HbA1c), high-sensitivity C-reactive protein (hsCRP), and a lipid panel. Serology testing was done at Quest Laboratories (Dallas, TX, USA).

### Statistics

Statistical analysis was performed using SigmaPlot 12.5 software (Systat Software, Inc., San Jose, CA, USA). Normality was assessed using a Shapiro-Wilk test. For those outcome measures with a normal distribution, data is represented as mean ± standard deviation. Significance between two groups was determined using a Student’s t-test. For outcome measures with a nonnormal distribution, data is presented as the median value with the range (min–max). Significance between two groups was determined using a Mann-Whitney rank sum test. Nominal variables were evaluated with a *χ*^2^ test. Pearson’s correlation analysis was used to determine a relationship between cup-to-disc ratio, low SpO2 and the apnea-hypopnea index (AHI). Statistical significance was set at *P* < 0.05.

## Results

### Participant demographics

A total of 67 participants were recruited for this study, 39 in the OSA group and 28 in the control group. Two participants within the control group had STOP BANG scores ≥5 and thus were excluded from analysis due to the possibility of having undiagnosed OSA. Two participants in the OSA group were removed due high hA1c levels (>6.5%) and one participant was determined not to have OSA upon review of their polysomnogram. Using the AHI to classify the severity of OSA, 64.5% of participants had mild OSA, 19.4% had moderate OSA, and 16.1% had severe OSA. Using the lowest oxygen saturation (SpO2) to classify OSA, 3.4% of participants had mild OSA, 55.2% had moderate OSA, and 41.4% had severe OSA. Participant characteristics are listed in Table 1. There was no significant difference in patient age between groups (60.3 years ± 14.8 years compared to 59.9 ± 12.5 years, P = 0.900, t-test). There was also no significant difference between sex (52.8% males and 47.2% females compared to 53.8% males and 46.2% females, P = 0.861, chi-square test). Similarly, race, ethnicity, and smoking status were not statistically different between groups (P = 0.151, P = 1.000, and P = 0.641, chi-square (race/ethnicity) and Fisher Exact (smoking) tests, respectively).

**Table 1:** Patient characteristics.

	OSA	Control	P value
	N = 36	N = 26	
**Age (years)**			
Mean ± SD	60.3 ± 14.8	59.9 ± 12.5	P = 0.900
Range	26–83	39–81	
**Sex n(%)**			
Male	19 (52.8%)	14 (53.8%)	P = 0.861
Female	17 (47.2%)	12 (46.2%)	
**Race n(%)**			
Black	8 (22.2%)	7 (26.9%)	P = 0.151
Caucasian	27 (75.0%)	15 (57.7%)	
Asian	1 (2.8%)	4 (15.4%)	
**Ethnicity n(%)**			
Hispanic/Latino	2 (5.6%)	1 (3.8%)	P = 1.000
Non-Hispanic/Latino	34 (94.4%)	25 (96.2%)	
**Smoker n(%)**			
Yes	2 (5.6%)	3 (11.5%)	P = 0.641
No	34 (94.4%)	23 (88.5%)	

Differences in numerical variables were assessed using a Student’s t-test. Differences in sex, race, and ethnicity were assessed using a Chi-square test and smoking status using a Fisher Exact test.

### Anthropometric measurements and serologies

Anthropometric measurements and serologies are listed in Table 2. The average BMI in both groups was in the obese range, although participants in the OSA group had higher BMI than the controls ((35.3, range 23.3–70.7) and (30.2, range 18.9–41.4), P = 0.008, Mann-Whitney rank sum test). Similar to BMI, waist circumference (P = 0.002, t-test), waist:height ratio (P = 0.029, Mann-Whitney rank sum test), and STOP-BANG scores (P < 0.001, Mann-Whitney rank sum test) were also increased in the OSA group. Neck circumference trended higher in the OSA group, but was not significantly different (P = 0.082, t-test). Systolic and diastolic blood pressures were elevated in both groups but were not statistically different (140.5 (98–231) mmHg systolic and 85.5 (56–124) mmHg diastolic compared to 133.5 (115–181) mmHg systolic and 87.5 (60–112) diastolic, P = 0.321 and P = 0.836, respectively, Mann-Whitney rank sum test). Mean HbA1C levels were 5.6 ± 0.4 in the OSA group and 5.7 ± 0.4 in the control group with no significant differences (P = 0.351, t-test). There were no significant differences in hsCRP, high density lipoproteins (HDL), triglycerides, or cholesterol to HDL ratio (P = 0.423, P = 0.242, P = 0.578, P = 0.954, respectively (t-test for hsCRP, Mann-Whitney rank sum test for all other measurements). However, the median value for low density lipoproteins was slightly but significantly higher in the control group (97.5 (range 56–208) compared to 112.0 (range 74.0–176.0), P = 0.019, Mann-Whitney rank sum test).

**Table 2:** Anthropometric measurements and serologies.

	OSA	Control	P value
	N = 36	N = 26	
**BMI**	35.3 (23.3–70.7)	30.2 (18.9–41.4)	*P = 0.008
**Waist circumference (in)**	43.8 ± 6.0	39.2 ± 4.7	*P = 0.002
**Waist:Height ratio**	0.7 (0.5–0.9)	0.6 (0.4–0.7)	*P = 0.029
**Neck circumference (in)**	15.5 ± 1.6	15.2 ± 1.4	P = 0.082
**Systolic blood pressure (mmHg)**	140.5 (98–231)	133.5 (115–181)	P = 0.321
**Diastolic blood pressure (mmHg)**	85.5 (56–124)	87.5 (60–112)	P = 0.836
**HbA1c (%)**	5.6 ± 0.4	5.7 ± 0.4	P = 0.351
**hsCRP (mg/L)**	2.3 (0.2–9.3)	2.0 (0.2–7.4)	P = 0.423
**HDL (mg/dL)**	50.5 (8–97)	51.5 (36–119)	P = 0.242
**Triglycerides (mg/dL)**	133.0 (55–323)	111.5 (62–380)	P = 0.578
**Chol/HDL**	3.6 (1.9–13.4)	3.9 (2.2–5.9)	P = 0.954
**LDL (mg/dL)**	97.5 (56–208)	112.0 (74–176)	*P = 0.019
**STOP-BANG score**	5.0 (2–8)	2.0 (0–4)	*P < 0.001

Normally distributed data are expressed as mean ± standard deviation. For data with a nonnormal distribution, data are expressed as median with range (min–max).

*Statistically significant, Student’s t-test or Mann-Whitney Rank Sum test.

Cornea and ocular surface metrics

Cornea and ocular surface metrics are listed in Table 3. There were no differences in tear production (P = 0.471), corneal fluorescein staining (P = 0.105) or conjunctival lissamine green staining (P = 0.625) staining between OSA participants and controls. For both groups, median tear secretion was consistent with a non-dry eye subject. While there were no differences in TFBUT between groups (P = 0.207, Mann-Whitney rank sum test), both exhibited TFBUTs less than 5 seconds, indicative of an unstable tear film. The OSDI score was the only dry eye test parameter that differed between groups, with a median value of 12.5 (range 0–70.8) in the OSA group compared to 8.7 (range 0–41.7) in the control group (P = 0.041, Mann-Whitney rank sum test). While some dry eye was evident between groups, there were no differences in the clinical signs of dry eye and only a small difference was found in patient reported symptoms.

**Table 3:** Cornea and ocular surface metrics.

	OSA	Control	P value
	N = 36	N = 26	
**BCVA (logMAR)**			
** OD**	0.11 (0–0.62)	0.01 (-0.12–0.34) ± 0.10	*P < 0.001
** OS**	0.08 (0–0.42)	0.01 (-0.12–0.28)	*P = 0.003
**TFBUT (sec)**	3.0 (1.4–30.0)	4.2 (1.3–28.9)	P = 0.207
**Schirmers (mm)**	12.3 (4.0–32.5)	16.5 (4.0–30.0)	P = 0.471
**NaFl staining**	1.8 (0–5.5)	1.0 (0–7.5)	P = 0.105
**LG staining**	2.8 (0–10)	2.0 (0–7)	P = 0.625
**OSDI score**	12.5 (0–70.8)	8.7 (0–41.7)	*P = 0.041
**Nerve fiber length (μm)**	1814.3 ± 476.5	1781.7 ± 543.8	P = 0.806
**Nerve fiber density (#/μm** ^**2**^ **)**	4.0 ± 1.2	4.1 ± 1.1	P = 0.625
**Nerve branch points (#)**	3.5 (1.5–11.0)	4.8 (0.7–14.8)	P = 0.332
**Dendritic cells (#)**	5.3 (0–35.0)	8.5 (1.0–27.5)	P = 0.243
**Corneal sensitivity (mm)**	45.0 (20.0–60.0)	45.0 (25.0–60.0)	P = 0.244

Normally distributed data are expressed as mean ± standard deviation. For data with a nonnormal distribution, data are expressed as median with range (min–max). *Statistically significant, Student’s t-test.

**Fig 1:**
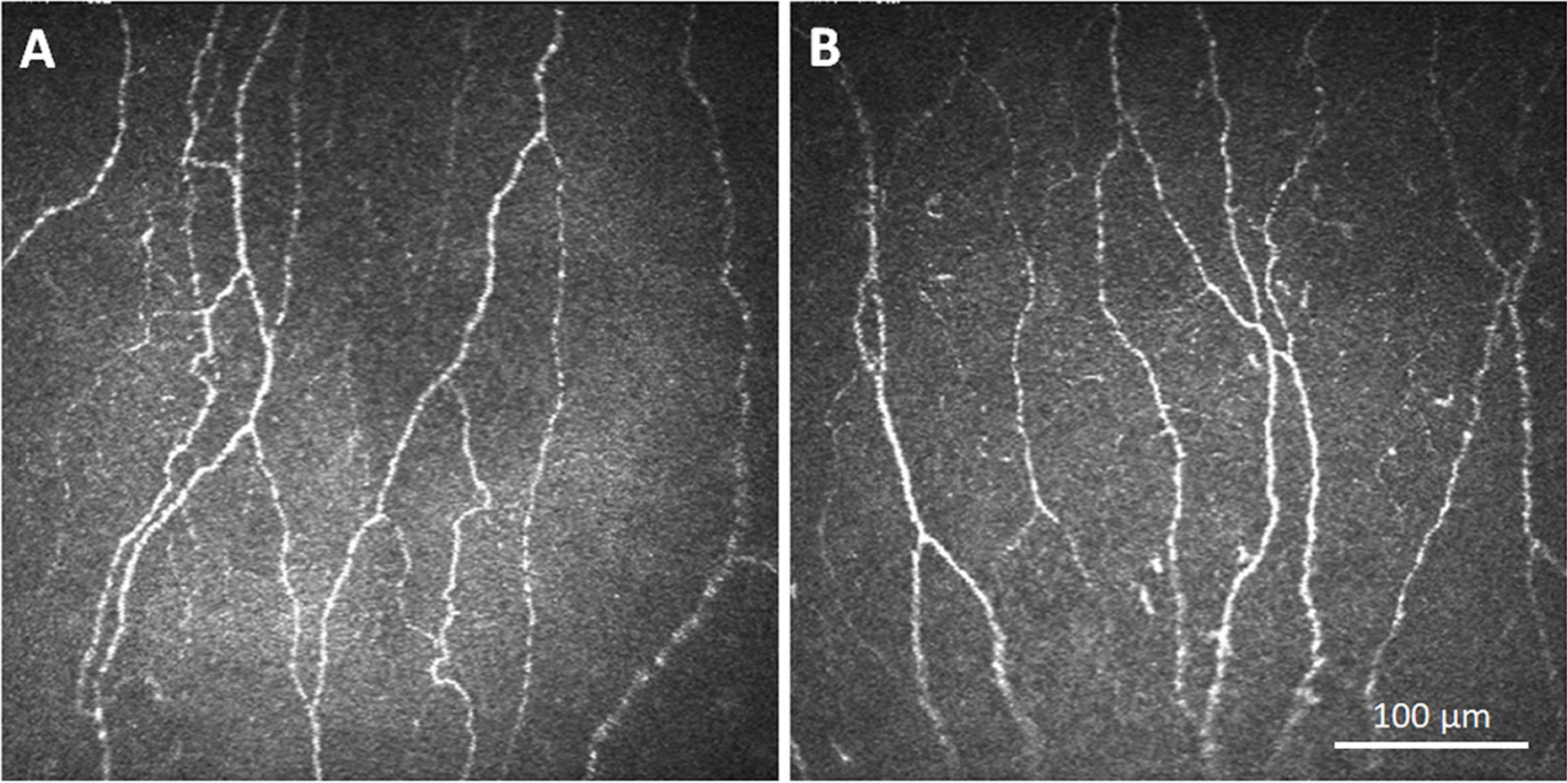
*In vivo* confocal images of the corneal subbasal nerve plexus (SBNP). (A) Confocal imaging showing a normal distribution of corneal subbasal nerve fibers in a patient with OSA. (B) Confocal image of the corneal subbasal nerve plexus in a non-OSA control patient. Scale bar: 100 μm.

In terms of corneal nerve structure and function, there were no differences in any of the test parameters (Table 3). Representative IVCM images of the SBNP are shown in Fig 1. The mean corneal nerve fiber length, mean corneal nerve fiber density, and mean corneal nerve fiber branch points were slightly higher in the control group with no significant differences between groups (P = 0.806, P = 0.625, P = 0.332, respectively). Similar to this, the number of dendritic cells was slightly, but not significantly higher in the control group (P = 0.243, Mann-Whitney). There was also no detectable difference in corneal sensitivity between groups. The median corneal sensitivity in both the OSA group and controls was 45.0 (P = 0.244, Mann-Whitney rank sum test).

### Retinal nerve fiber layer thickness

**Fig 2:**
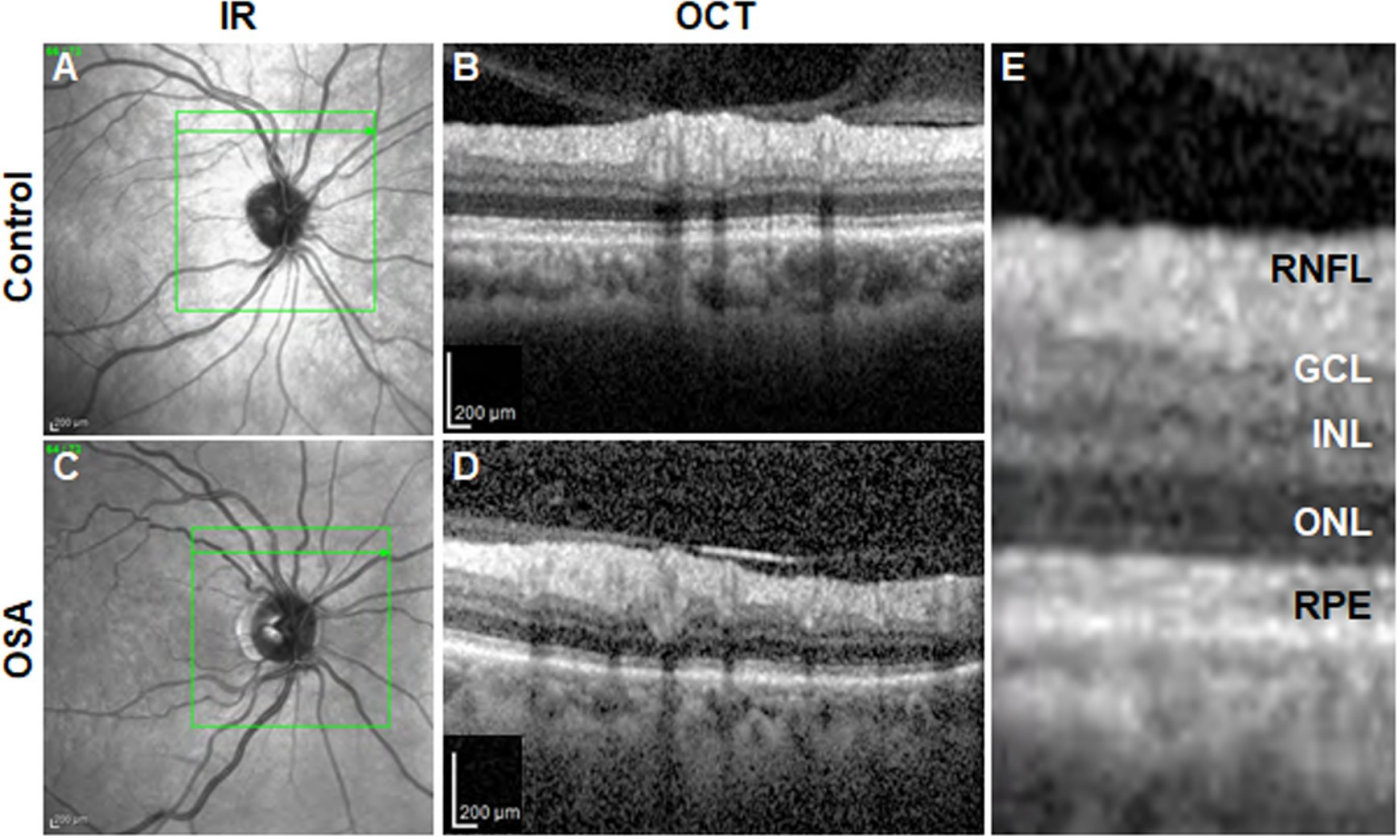
Retinal cell layers visible within the retinal nerve fiber layer (RNFL) scans. Each patient underwent multiple scans around the optic nerve head to obtain morphological information of the retinal cell layers in this region. (A) Representative infrared (IR) image depicting the region where the scans were collected (green square) in a control patient. (B) Representative optical coherence tomography (OCT) image from the control patient highlighting one measurement (green arrow in A) collected for examining the retinal morphology. (C) Representative IR image depicting the region where the scans were collected (green square) in a patient with OSA. (D) Representative OCT image from the OSA patient highlighting one measurement (green arrow in C) collected for examining the retinal morphology. (E) High magnification OCT image from B with all retinal cell layers identified. GCL, ganglion cell layer; INL, inner nuclear layer; ONL, outer nuclear layer; RPE, retinal pigment epithelium. Scale bar: 200 μm.

**Fig 3:**
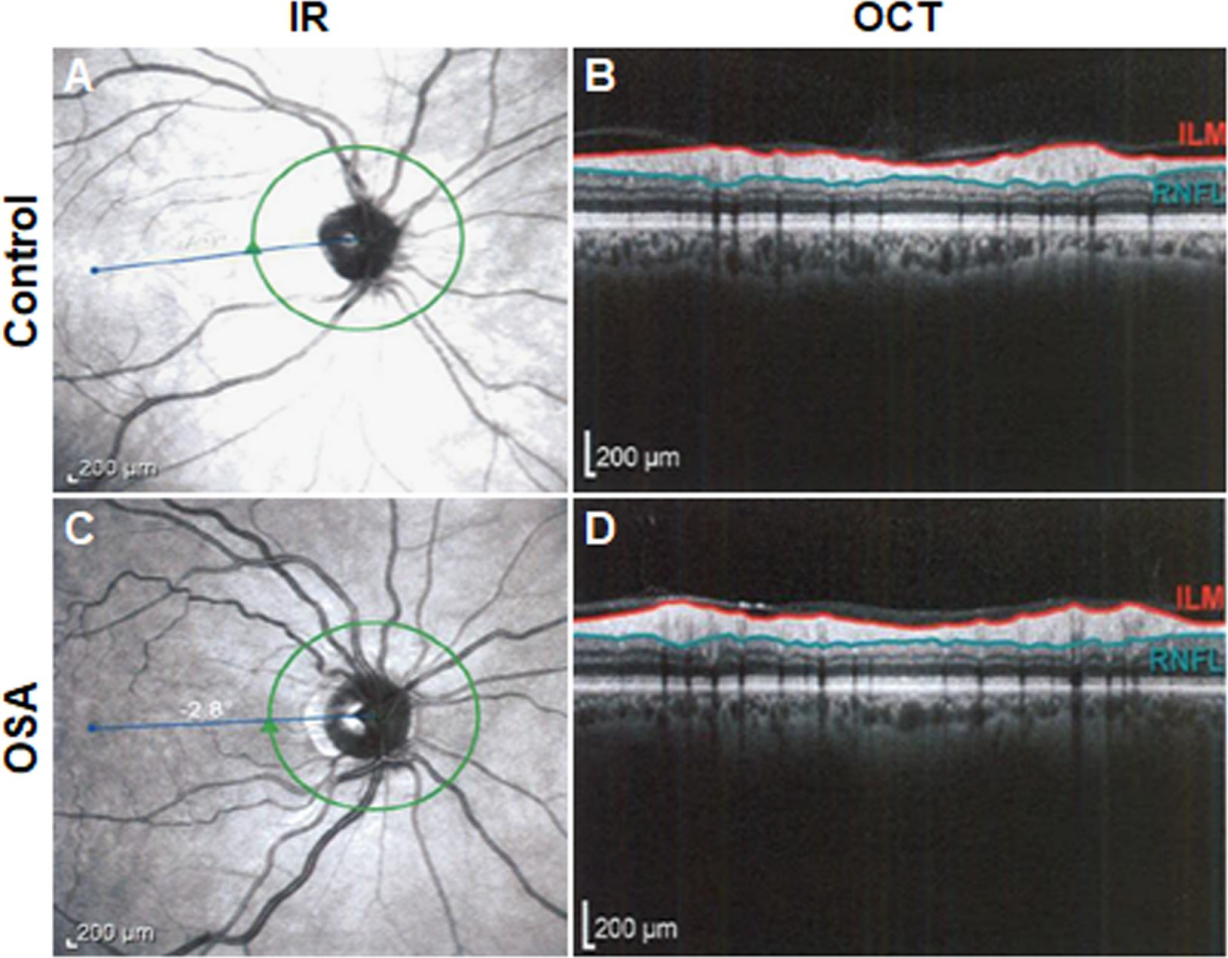
Quantification of the RNFL. The data presented in Tables 4 and 5 were obtained through multiple measurements around the optic nerve head (green circle) recording the thickness between the inner limiting membrane (ILM; red) and the RNFL (blue). (A) Representative infrared (IR) image depicting the region where the scans were collected in a control patient. (B) Representative optical coherence tomography (OCT) image from the control patient highlighting one measurement collected for thickness quantification between the ILM and RNFL. (C) Representative IR image depicting the region where the scans were collected in a patient with OSA. (D) Representative OCT image from the control patient highlighting one measurement collected for thickness quantification between the ILM and RNFL. Scale bar: 200 μm.

RNFL thickness measurements, measured at the optic disc/peripapillary region, are shown in Table 4. Representative images of the visible cell layers and region of measurement are shown in Figs 2 and 3. Thinning of the RNFL was significant in the inferior quadrant (114.2 μm ± 19.0 μm compared to 127.3 μm ± 17.3 μm, P = 0.008, t-test). Within the inferior quadrant, significant thinning was seen in the inferior temporal sector (129.0 μm ± 22.7 μm compared to 142.0 μm ± 21.5 μm, P = 0.028, t-test) and the inferior nasal sector (99.8 μm ± 25.7 μm compared to 114.4 μm ± 26.7 μm, P = 0.036, t-test). The superior quadrant of the RNFL was also somewhat thinner in the OSA group, (110.7 μm ± 16.4 μm compared to 120.0 μm ± 14.4 μm, P = 0.048, t-test). When the superior quadrant was further subdivided into superior nasal and superior temporal sectors, significant thinning was evident in the superior nasal sector (96.4 μm ± 20.9 μm compared to 107.7 μm ± 20.1 μm, P = 0.04, t-test). While there was a trend toward a decrease in the mean thickness of the superior temporal sector in the OSA group, this finding was not significantly different (124.8 μm ± 21.9 μm compared to 131.9 μm ± 15.4 μm, P = 0.304, t-test). There were no differences between the temporal and nasal quadrants (P = 0.136 and P = 0.313, respectively, t-test). The global average was significantly decreased in the OSA group (P = 0.022, t-test). OCT scans were also acquired of the macular to obtain measurements of total retinal thickness in this region. Representative images of the visible cell layers and region of measurement are shown in S2 and S3 Figs. Global macular thickness was not significantly different in the OSA group compared to the control (277.5 μm ± 226.5 μm and 275.2 μm ± 28.7 μm, respectively, t-test). There were no differences in macular thickness in any of the quadrants (S4 Fig).

**Table 4:** Retinal nerve fiber layer thickness (μm).

	OSA	Control	P value
	N = 36	N = 26	
**Superior**	110.7 ± 16.4	120.0 ± 14.4	*P = 0.048
**Superior temporal**	124.8 ± 21.9	131.9 ± 15.4	P = 0.304
**Superior nasal**	96.4 ± 20.9	107.7 ± 20.1	*P = 0.04
**Nasal**	68.7 ± 12.8	72.6 ± 16.9	P = 0.313
**Inferior**	114.2 ± 19.0	127.3 ± 17.3	*P = 0.008
**Inferior temporal**	129.0 ± 22.7	142.0 ± 21.5	*P = 0.028
**Inferior nasal**	99.8 ± 25.7	114.4 ± 26.7	*P = 0.036
**Temporal**	64.8 ± 11.3	69.1 ± 10.3	P = 0.136
**Global average**	89.7 ± 10.4	97.3 ± 9.0	*P = 0.022

Normally distributed data are expressed as mean ± standard deviation.

*Statistically significant, Student’s t-test.

**Table 5:** Risk factors for glaucoma and need for referral to a glaucoma specialist.

	OSA	Control	P value
	N = 36	N = 26	
**Glaucoma referral n (%)**			
Yes	15 (41.7%)	3 (11.5%)	*P < 0.022
No	21 (58.3%)	23 (88.5%)	
**Cup-to-disc ratio**	0.3 (0.2–0.7)	0.3 (0.15–0.7)	**P = 0.012
**Family history n (%)**			
Yes	11 (30.6%)	6 (23.1%)	P = 0.774
No	22 (61.1%)	17 (65.4%)	
Unknown	3 (8.3%)	3 (11.5%)	
**Low SpO**_**2**_	81.4% (65.0–91.0)	---	N/A
**Apnea Hypopnea Index**	18.7 (5.2–91.5)	---	N/A
**Severity n(%)**		---	N/A
Mild	20 (64.5%)		
Moderate	6 (19.4%)		
Severe	5 (16.1%)		

Normally distributed data are expressed at mean ± standard deviation. For data with a nonnormal distribution, data are expressed as median with range (min–max).

*Statistically significant, Chi-square test

**Mann-Whitney Rank Sum test.

**Fig 4:**
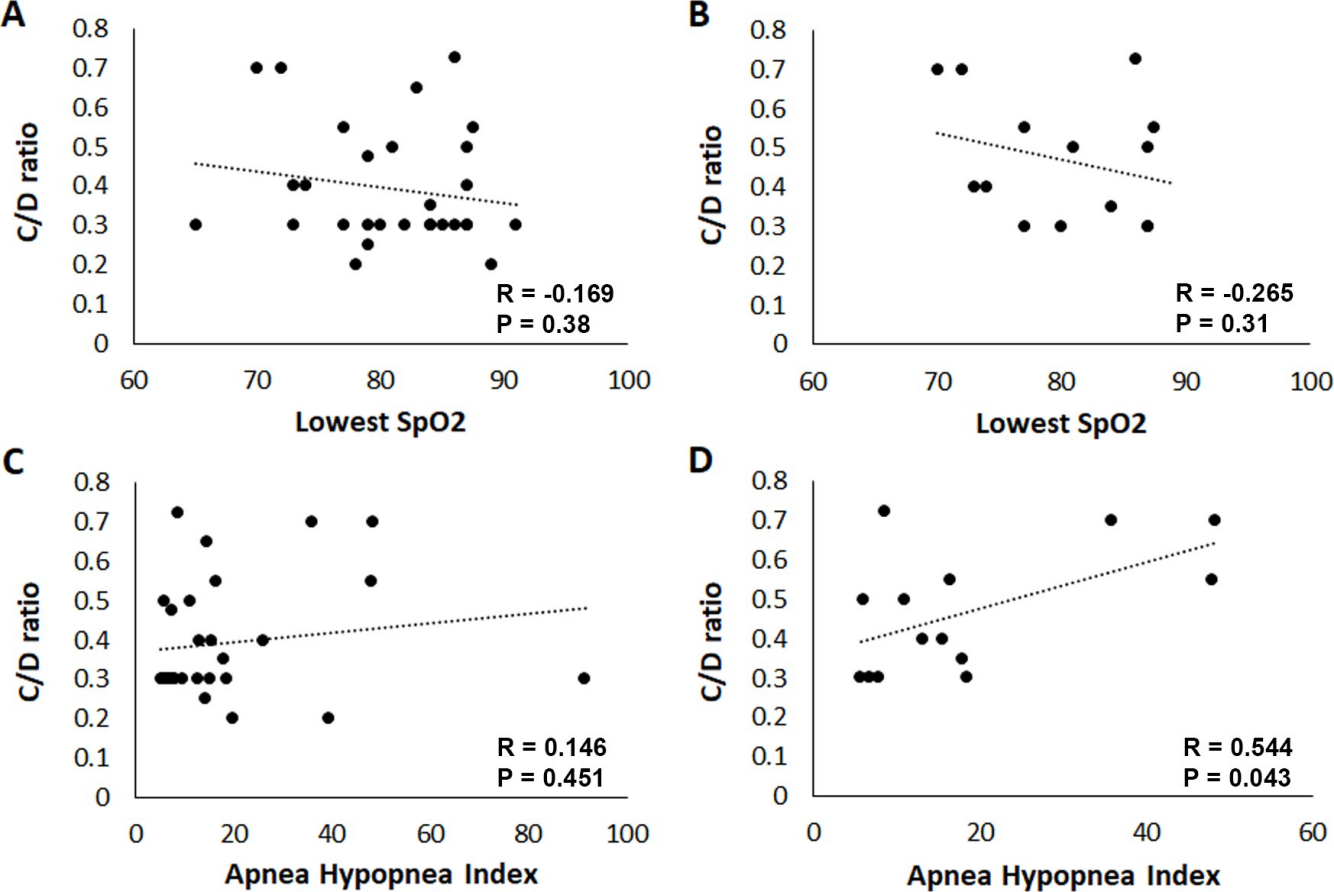
The relationship between OSA parameters and cup-to-disc ratio. (A) There was no correlation between the lowest SpO2 value and the cup-to-disc ratio for all patients in the OSA group (N = 36, R = -0.169, P = 0.38). (B) Similarly, there was no correlation between the lowest SpO2 value and the cup-to-disc ratio for patients that were referred for a glaucoma evaluation (N = 15, R = -0.265, P = 0.31). (C) There was no correlation between the apnea hypopnea index and the cup-to-disc ratio in all patients in the OSA group (N = 36, R = 0.146, P = 0.451). (D) There was a significant positive correlation between the apnea hypopnea index and the cup-to-disc ratio for patients that were referred for a glaucoma evaluation (N = 15, R = 0.546, P = 0.043).

Due to the characteristic pattern of RNFL thinning in the OSA group, we further evaluated their cup-to-disc ratio. While the median value for the cup-to-disc ratio was the same between the OSA group and controls, control patients on average had a smaller cup-to-disc ratio (P = 0.012, Mann-Whitney rank sum test). Within the OSA group, a greater percentage of participants were referred for a glaucoma evaluation due to an abnormal optic nerve exam. A total of 41.7% of participants within the OSA group were referred for subsequent evaluation compared to 11.5% of participants in the control group (P = 0.022, chi-square test). There were no differences in the number of patients that had a known family history for glaucoma between groups (P = 0.774, chi-square). Correlations were then investigated between participants with atypical optic nerves that were referred for a glaucoma workup. When all participants in the OSA group were analyzed, there was no correlation between the lowest SpO2 and cup-to-disc ratio (R = -0.169, P = 0.38, Fig 4A). Similarly, for patients with OSA that were referred for a glaucoma exam, there was no correlation between cup-to-disc ratios and lowest SpO2 (R = -0.265, P = 0.31, Fig 4B). When compared against the apnea hypopnea index, there was no correlation between cup-to-disc and AHI for all OSA participants (R = 0.146, P = 0.451, Fig 4C). However, a comparison of the cup-to-disc ratio and AHI for OSA participants that were referred for a glaucoma evaluation revealed a moderate correlation (R = 0.546, P = 0.043, Fig 4D and S5 Fig). A chart review was completed for the 15 participants who underwent a complete glaucoma workup for an abnormal optic disc. At that visit, mean intraocular pressure was 14.4 mmHg within the OSA group (range 11–17 mmHg). Eight (53.3%) participants were diagnosed as glaucoma suspects, 1 (6.7%) participant was diagnosed with optic rim pallor, 1 (6.7%) participant had a tilted optic disc that resulted in thin OCT measurements, and 5 (33.3%) participants were lost to follow up.

## Discussion

In this study, we evaluated the effect of OSA on the corneal SBNP and the RNFL. Our first key finding was the presence of RNFL thinning in the optic nerve/peripapillary region in participants with OSA. RNFL thinning has been previously reported in patients with OSA, along with decreased visual evoked potentials [9, 10, 31–47, 56]. Importantly, while RNFL thinning has been reported in myopic eyes and associated with health conditions including migraines, diabetes, multiple sclerosis, Parkinson’s disease, and Alzheimer’s disease, this usually results in more diffuse thinning [57–62]. In contrast, the pattern of thinning shown in this study is consistent with glaucomatous changes that follow an ISNT (inferior, superior, nasal, and then temporal) pattern. This finding is in agreement with two recent meta-analyses that demonstrated overall thinning of RNFL, with the greatest amount of thinning within the inferior and superior quadrants [46, 47]. Due to the pattern of RNFL thinning, we further investigated the effect of OSA on the cup-to-disc ratio. Overall, patients with OSA had larger cup-to-disc ratios than control participants. The cup-to-disc ratio was not associated with OSA severity when using either the lowest oxygen saturation or the apnea-hypopnea index as the independent variable. However, after subdividing OSA patients according to those that required a referral to a glaucoma specialist for additional testing and those that had healthy, non-glaucomatous nerves, the apnea-hypopnea index was correlated with increased cupping of the optic nerve. Together, these findings suggest that in patients with suspicious optic nerve heads, the magnitude of the cup-to-disc ratio is increased in patients with more severe OSA.

The pathophysiology behind RNFL thinning in OSA is unknown. It has been widely hypothesized that the etiology involves hypoxic and vascular changes in the absence of increased intraocular pressure [41, 63, 64]. Several different physiologic mechanisms have thus been proposed. Prolonged apneas and hypopneas result in hypoxia, with sympathetic activation causing hypertension and increased vascular resistance [65]. This dysregulation leads to autonomic dysfunction, with the potential to disturb blood flow to the optic disc [66]. Endothelial damage and vascular dysregulation occur in OSA due to oxidative damage, inflammation, and an imbalance between vasodilators and vasoconstrictors [5]. Likewise, during sleep, there is an increase in intracranial pressure that in turn decreases cerebral perfusion pressure. The decreased cerebral perfusion pressure has been hypothesized to result in poor blood flow to the optic disc and RNFL [42, 67, 68]. Indeed, studies have shown a direct decrease in optic nerve blood supply and thinning of the choroidal vasculature in OSA [31, 32, 44, 48, 69].

Despite anatomical differences in innervation, certain systemic diseases have been associated with both RNFL thinning and damage to the corneal SBNP. With respect to the latter, loss of the corneal SBNP has been shown to precede the onset of diabetic peripheral neuropathy and has been well documented in patients with small fiber neuropathy due to various underlying diseases [21]. In animal studies, systemic treatment to inhibit diabetes-induced vasoconstriction of arteries and arterioles has been shown to block loss of terminal nerve fibers in the hindpaw epidermis and in the corneal epithelium, and restores corneal sensitivity [30]. Since OSA is associated with micro and macro vascular damage, including an increase in endothelin, a potent vasoconstrictor, this study investigated whether the cornea was similarly affected [70]. This led to our second key finding, the absence of any detectable effects on corneal nerve fiber morphology or sensitivity in this cohort. It should be noted however, that participants were not screened for the presence of peripheral neuropathy or evidence of intraepidermal nerve fiber loss. In addition, while confocal microscopy is able to detect SBNP changes in diabetic subjects prior to the onset of peripheral neuropathy, the underlying mechanism(s) that contribute to the development of neuropathy likely differ between diabetes and OSA. It is possible that the participants in this study may have been too early in the disease process for quantifiable changes to be seen. Moreover, for inclusion, polysomnograms were only required to have been obtained within a five-year period and a host of systemic and anthropometric variables, including weight, may have changed since diagnostic testing occurred.

It is well documented that use of certain masks in participants with OSA can promote corneal damage due to continuous air flow leaking from the mask and blowing on the eye during sleep. In the present study, we excluded participants that were compliant with CPAP and those with floppy eyelid syndrome to control for negative interactions on the ocular surface that may have impacted our outcome measures [71]. CPAP therapy was further excluded as this would have also introduced a treatment arm into the study. While there was evidence of dry eye disease in both cohorts, groups were matched for clinical signs of dry eye disease. Thus, this allowed us to control for the effects of chronic inflammation on the ocular surface.

In summary, this works suggests that OSA differentially affects the RNFL and corneal SBNP. It further indicates that patients presenting with OSA and glaucomatous changes in the optic nerve head should be carefully monitored, as an increase in OSA severity, based on the apnea-hypopnea index, is associated with increased optic nerve cupping. While OSA does not appear to exert a robust effect on corneal nerves, it does not fully exclude a relationship between OSA and the onset of corneal neuropathy. In contrast to diabetes where corneal nerve changes represent a sensitive early metric for the onset of peripheral neuropathy, this may not be the case for OSA. Thus, future studies need to evaluate participants with evidence of existing peripheral neuropathy. Likewise, overnight polysomnogram data at the time of testing, including those within the healthy control group, may be necessary. Although the use of the STOP-BANG questionnaire to screen for risk of OSA in the present study has been well validated, it does not replace the polysomnogram as a diagnostic modality [72, 73].

## Supporting information

S1 Fig CPAP compliance survey. (DOCX)

S2 Fig Retinal cell layers within the macular scans. Each patient underwent multiple scans of the macular region to obtain morphological information of the retinal cell layers within this region. (A) Representative infrared (IR) image depicting the region where the scans were collected (green lines) in a control patient. (B) Representative optical coherence tomography (OCT) image from the control patient highlighting one measurement (green arrow in A) collected for examining the retinal morphology. (C) Representative IR image depicting the region where the scans were collected (green lines) in a patient with obstructive sleep apnea (OSA). (D) Representative OCT image from the OSA patient highlighting one measurement (green arrow in C) collected for examining the retinal morphology. (E) High magnification OCT image from B with all retinal cell layers identified. RNFL, retinal nerve fiber layer; GCL, ganglion cell layer; INL, inner nuclear layer; ONL, outer nuclear layer; RPE, retinal pigment epithelium.
(DOCX)

S3 Fig Quantification of macular thickness. The data presented in S4 Fig were obtained through multiple measurements around the macula (blue circle) recording the thickness between the inner limiting membrane (ILM) and the Bruch’s membrane (BM; red lines). (A) Representative infrared (IR) image depicting the region of the macula where the scans were collected in a control patient. Color heatmap represents thickness depth of the individual regions, with blue depicting reduced thickness and red increased thickness. (B) Representative optical coherence tomography (OCT) image from the control patient highlighting one measurement collected for thickness quantification between the ILM and BM (red lines). (C) Representative IR image depicting the region where the scans were collected in a patient with obstructive sleep apnea (OSA). Color heatmap represents thickness depth of the individual regions, with blue depicting reduced thickness and red increased thickness. (D) Representative OCT image from the control patient highlighting one measurement collected for thickness quantification between the ILM and BM (red lines).
(DOCX)

S4 Fig Macular thickness was unchanged in the OSA group compared to controls. Macular thickness was measured as described in S3 Fig. (A) Diagram showing the quadrants that were analyzed. (B) Central macular thickness at the fovea. (C) Inner macular layers. (D) Outer macular layers. S, superior quadrant; N, nasal quadrant; I, inferior quadrant; T, temporal quadrant. Data presented as mean ± standard deviation. T-test comparing OSA to control for each measurement. No significant differences were found.
(DOCX)

S5 Fig Sensitivity analysis confirming the relationship between OSA and increased optic nerve head cupping. (A) Participants in the OSA group that were referred for a subsequent glaucoma evaluation had a larger cup-to-disc ratio compared to participants with OSA that were not referred (P = 0.011, t-test). (B) A box and whisker plot showed outliers within the OSA subgroup that were not referred for a glaucoma evaluation. No outliers were present in the OSA subgroup that were referred. (C) After removing the outliers from the non-referred subgroup, the difference between the cup-to-disc ratio in OSA patients that were referred compared to those that were not referred was further increased (P < 0.001, t-test). Data presented as mean ± standard deviation. Yes, OSA patients that received a referral to the glaucoma service; No, OSA patients that did not receive a referral.
(DOCX)
