# Supplementary material for: Differential effects of obstructive sleep apnea on the corneal subbasal nerve plexus and retinal nerve fiber layer
Source: PLoS One. 2022 Jun 30;17(6):e0266483. doi: 10.1371/journal.pone.0266483 (PMC9246161; doi:10.1371/journal.pone.0266483)
Supplement: S1 Fig — (DOCX) [file pone.0266483.s001.docx]

**S1 Fig.**

**CPAP Compliance Questionnaire**

Subject Number: ____________

1. Has your doctor ever recommended a CPAP for treatment of obstructive sleep apnea?
   1. Yes
   2. No
2. Did you ever try to use the CPAP?
   1. Yes
   2. No

*If answer is no, you may stop here.

1. Did you begin using the CPAP and then stopped using it?
   1. Yes
   2. No
   3. If yes, how long since you have last tried it? _______________
2. If you use a CPAP, do you regularly use it less than 4 hours per night?
   1. Yes
   2. No
   3. If yes, how many hours (on average) do you use it? _________________
   4. How many nights per week (on average) do you use it? ________________
3. If you use a CPAP, do you regularly use it for more than 4 hours per night?
   1. Yes
   2. No
   3. If yes, how many hours (on average) do you use it? _________________
   4. How many nights per week (on average) do you use it? _________________
4. If you use a CPAP, what type of mask do you wear:
   1. Nasal pillows beneath nose
   2. Mask covering nose
   3. Full face mask covering nose and mouth
5. Do you ever experience any eye irritation from use of your CPAP (such as dryness, scratchiness or itching?
   1. Yes
   2. No
