# Supplementary figures and images for: Differential effects of obstructive sleep apnea on the corneal subbasal nerve plexus and retinal nerve fiber layer
Source: PLoS One. 2022 Jun 30;17(6):e0266483. doi: 10.1371/journal.pone.0266483 (PMC9246161; doi:10.1371/journal.pone.0266483)

**S2 Fig.**


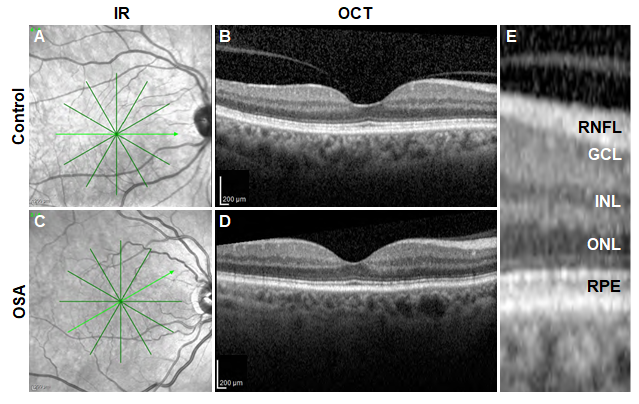

Supplement: S2 Fig — Each patient underwent multiple scans of the macular region to obtain morphological information of the retinal cell layers within this region. (A) Representative infrared (IR) image depicting the region where the scans were collected (green lines) in a control patient. (B) Representative optical coherence tomography (OCT) image from the control patient highlighting one measurement (green arrow in A) collected for examining the retinal morphology. (C) Representative IR image depicting the region where the scans were collected (green lines) in a patient with obstructive sleep apnea (OSA). (D) Representative OCT image from the OSA patient highlighting one measurement (green arrow in C) collected for examining the retinal morphology. (E) High magnification OCT image from B with all retinal cell layers identified. RNFL, retinal nerve fiber layer; GCL, ganglion cell layer; INL, inner nuclear layer; ONL, outer nuclear layer; RPE, retinal pigment epithelium. (DOCX) [file pone.0266483.s002.docx]

**S3 Fig.**


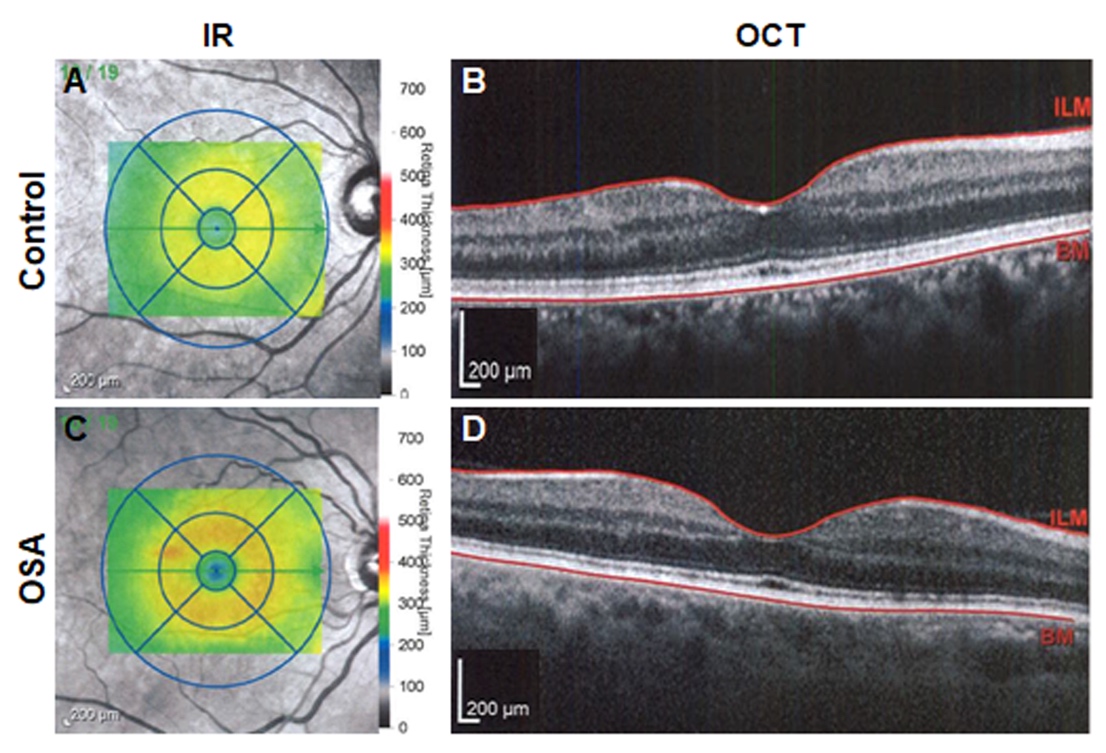

Supplement: S3 Fig — The data presented in S4 Fig were obtained through multiple measurements around the macula (blue circle) recording the thickness between the inner limiting membrane (ILM) and the Bruch’s membrane (BM; red lines). (A) Representative infrared (IR) image depicting the region of the macula where the scans were collected in a control patient. Color heatmap represents thickness depth of the individual regions, with blue depicting reduced thickness and red increased thickness. (B) Representative optical coherence tomography (OCT) image from the control patient highlighting one measurement collected for thickness quantification between the ILM and BM (red lines). (C) Representative IR image depicting the region where the scans were collected in a patient with obstructive sleep apnea (OSA). Color heatmap represents thickness depth of the individual regions, with blue depicting reduced thickness and red increased thickness. (D) Representative OCT image from the control patient highlighting one measurement collected for thickness quantification between the ILM and BM (red lines). (DOCX) [file pone.0266483.s003.docx]

**S4 Fig.**


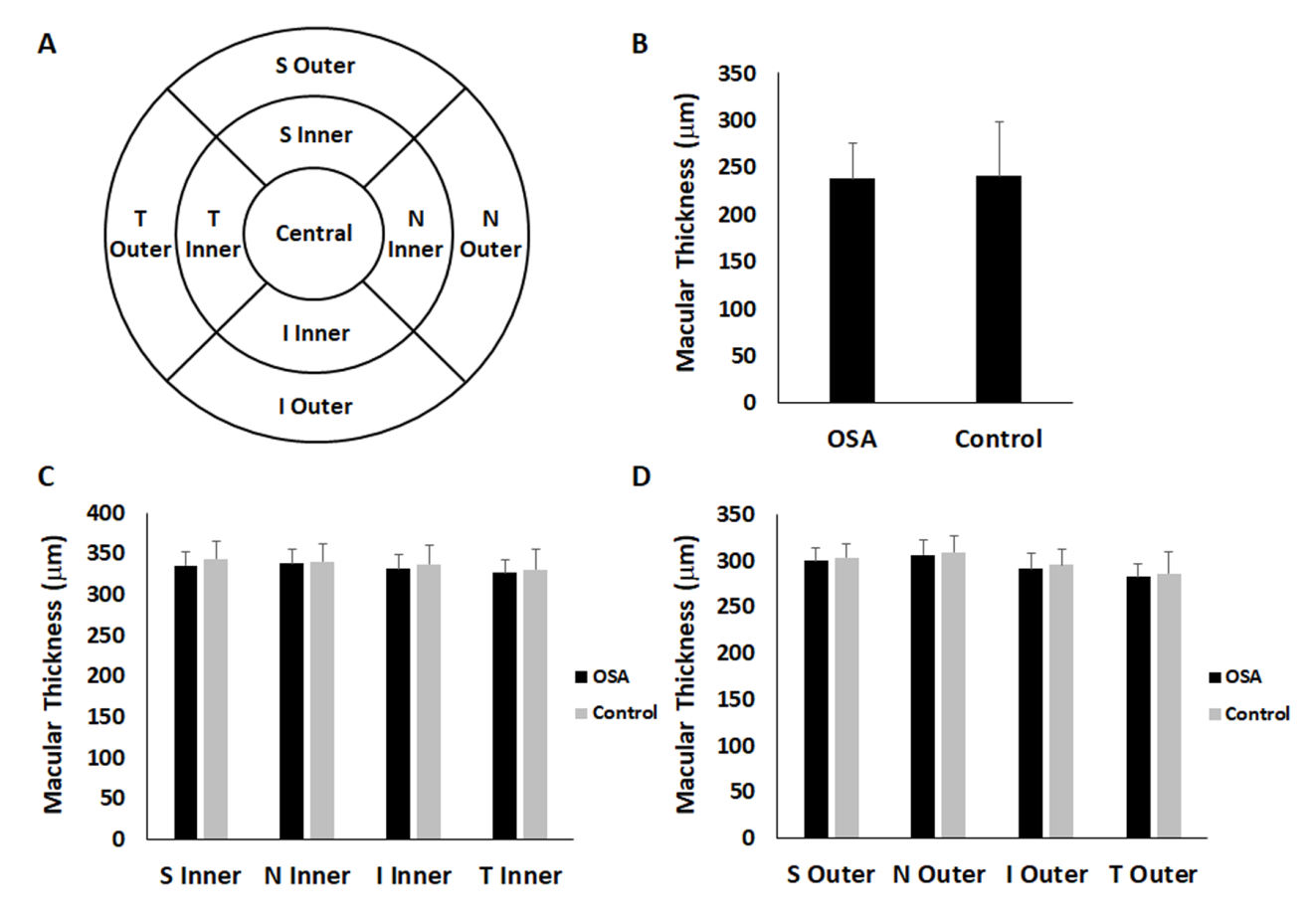

Supplement: S4 Fig — Macular thickness was measured as described in S3 Fig. (A) Diagram showing the quadrants that were analyzed. (B) Central macular thickness at the fovea. (C) Inner macular layers. (D) Outer macular layers. S, superior quadrant; N, nasal quadrant; I, inferior quadrant; T, temporal quadrant. Data presented as mean ± standard deviation. T-test comparing OSA to control for each measurement. No significant differences were found. (DOCX) [file pone.0266483.s004.docx]

**S5 Fig.**


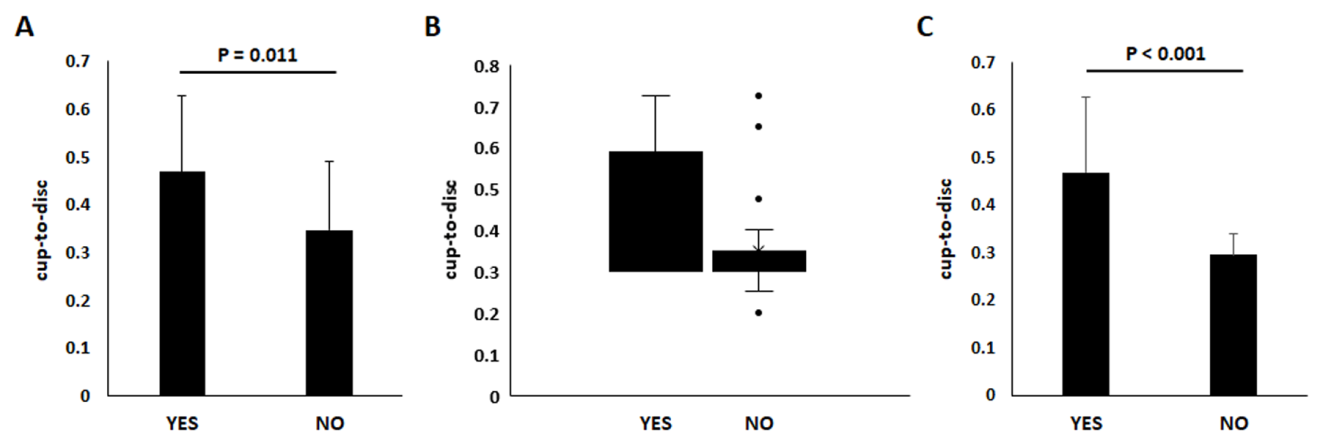

Supplement: S5 Fig — (A) Participants in the OSA group that were referred for a subsequent glaucoma evaluation had a larger cup-to-disc ratio compared to participants with OSA that were not referred (P = 0.011, t-test). (B) A box and whisker plot showed outliers within the OSA subgroup that were not referred for a glaucoma evaluation. No outliers were present in the OSA subgroup that were referred. (C) After removing the outliers from the non-referred subgroup, the difference between the cup-to-disc ratio in OSA patients that were referred compared to those that were not referred was further increased (P < 0.001, t-test). Data presented as mean ± standard deviation. Yes, OSA patients that received a referral to the glaucoma service; No, OSA patients that did not receive a referral. (DOCX) [file pone.0266483.s005.docx]
